# High-Intensity Warm-Up Increases Anaerobic Energy Contribution during 100-m Sprint

**DOI:** 10.3390/biology10030198

**Published:** 2021-03-05

**Authors:** Seung-Bo Park, Da-Sol Park, Minjun Kim, Eunseok Lee, Doowon Lee, Jaewoo Jung, Seong Jun Son, Junggi Hong, Woo-Hwi Yang

**Affiliations:** Graduate School of Sports Medicine, CHA University, Seongnam-si 13503, Gyeonggi-do, Korea; parks0524@naver.com (S.-B.P.); dasolpark@chauniv.ac.kr (D.-S.P.); minjunkim0113@gmail.com (M.K.); Eunseok4598@naver.com (E.L.); doowonlee1992@gmail.com (D.L.); jaewoojung0923@gmail.com (J.J.); seongjunson@gmail.com (S.J.S.); ptlhong@cha.ac.kr (J.H.)

**Keywords:** blood lactate, energetic contribution, glycolysis, phosphagen, maximal anaerobic performance

## Abstract

**Simple Summary:**

Certain exercise performances or movements cause sudden changes (or increases) in metabolic response. Track and field running events that require explosive energy in the shortest time, such as a 100-m sprint, need an immediate energy supply. Referring to the relevant studies to date, metabolic responses to submaximal exercise have been well documented, while information on the metabolic responses of short-term sprint performance is relatively insufficient. In this regard, based on the evidence that the human body relies on anaerobic energy metabolism during intense, short-term exercise, we investigated anaerobic energy contributions following the acute effect of a high-intensity warm-up during a 100 m-sprint. The main finding of our study revealed that a high-intensity warm-up (HIW) increases the contribution of the anaerobic system, probably by activating key regulatory enzymes related to anaerobic energy metabolism, compared to a low-intensity warm-up, for a 100-m sprint. Therefore, an HIW is effective in increasing anaerobic energy contribution during a 100-m sprint, which can be a useful strategy for coaches and athletes in the field.

**Abstract:**

This study aimed to evaluate the effects of warm-up intensity on energetic contribution and performance during a 100-m sprint. Ten young male sprinters performed 100-m sprints following both a high-intensity warm-up (HIW) and a low-intensity warm-up (LIW). Both the HIW and LIW were included in common baseline warm-ups and interventional warm-ups (eight 60-m runs, HIW; 60 to 95%, LIW; 40% alone). Blood lactate concentration [La^−^], time trial, and oxygen uptake (VO_2_) were measured. The different energy system contribution was calculated by using physiological variables. [La^−1^]_Max_ following HIW was significantly higher than in LIW (11.86 ± 2.52 vs. 9.24 ± 1.61 mmol·L^−1^; *p* < 0.01, respectively). The 100-m sprint time trial was not significantly different between HIW and LIW (11.83 ± 0.57 vs. 12.10 ± 0.63 s; *p* > 0.05, respectively). The relative (%) phosphagen system contribution was higher in the HIW compared to the LIW (70 vs. 61%; *p* < 0.01, respectively). These results indicate that an HIW increases phosphagen and glycolytic system contributions as compared to an LIW for the 100-m sprint. Furthermore, an HIW prior to short-term intense exercise has no effect on a 100-m sprint time trial; however, it tends to improve times (decreased 100-m time trial; −0.27 s in HIW vs. LIW).

## 1. Introduction

The question of how specific energy-providing pathways, induced by anaerobic metabolism, affect short-term intense exercise is a subject of growing interest to exercise physiologists [1]. In particular, the 100-m sprint in outdoor track and field running events utilizes immediate energy production for synthesizing adenosine triphosphate (ATP), predominantly via the use of phosphagen and glycolytic systems [2]. According to a previous study, the percentage of the anaerobic system utilized for this purpose (i.e., phosphagen and glycolytic system) is approximately 90~95% for 100-m sprint running (~10 s) [3,4].

Phosphagen, as the fastest and most powerful substrate energy system, contributes predominantly to speed acceleration during the first three seconds (approximately) [5,6,7]. In the phosphagen system, the creatine kinase reaction is based heavily on the concentration of intramuscular stores of phosphocreatine (PCr), since, to provide ATP, only one metabolic reaction is required during the first few seconds [8,9]. Hirvonen et al. [7] reported that maximum sprint performance relies on the capacity of an athlete to catalyze high-energy phosphates, as elite sprinters (100-m) demonstrated a superior ability to breakdown PCr. However, since skeletal muscle PCr stores are limited, increasing the relative contribution of glycolytic energy metabolism is important as maximal running speed starts to decline [10,11,12,13].

The most important aspect of the phosphagen system, particularly the adenylate kinase reaction, is the production of adenosine monophosphate (AMP), which is a potent allosteric activator of two enzymes [14]. AMP stimulates phosphorylase, which activates glycogenolysis and, in turn, offers immediate fuel for glycolysis, as well as activating phosphofructokinase (PFK) [8,14]. PFK is a key regulatory enzyme in the glycolytic pathway, and its activation facilitates an increased ATP regeneration rate [8,14]. In this regard, previous studies have reported that the glycolytic system supplied 50~55% of the energy demands during a 100-m sprint lasting approximately 10 s [15,16]. Therefore, the rapid activation of the glycolytic pathway also plays a key role in 100-m sprint performance, especially in the deceleration phases [4,7,12].

The optimal glycolytic capacity relies essentially on the amount of PFK [17], as it can be measured based on the metabolite lactate and the maximum work-load attained [18]. A marker of the glycolytic system is the accumulated lactate concentration following an intense short-term exercise [5,17,19]. Athletes with relatively poor anaerobic capacity were observed to have reduced PFK activity, as the concentration of hydrogen electrons increases during maximum exercise [12,18,20]. In light of this, even if the adenylic acid system is massively depleted, the rapid formation of lactate is no longer possible [14,17]. Thus, the maximum glycolytic capacity can be considered by increases in the concentration of blood lactate after short-term intense exercise [5,18,21,22].

In a review of previous studies on anaerobic performance, a high-intensity warm-up (HIW) was associated with increased effectiveness for short-term sprint performance [23,24,25,26]. Several studies have reported that short-term intense exercise induces an increase in cytosolic adenosine diphosphate (ADP), AMP, and inorganic phosphate (P_i_) triggered by the reactions of the glycolytic pathway, with the help of more chemical energy from the high-energy phosphate pool [8,14,27,28]. This phenomenon implies that HIW induces a higher rate of anaerobic enzyme activity that may affect the proportional contribution of phosphagen and glycolytic systems in 100-m sprinting. However, there have been no studies that separately investigate the anaerobic energy contributions following the acute effect of HIW during a 100-m sprint.

In the present study, to analyze how warm-up intensity can affect the contribution of anaerobic energy components during a 100-m sprint, physiological parameters were evaluated (blood lactate concentration, oxygen consumption, and heart rate) and contributions of the three energy systems were calculated (phosphagen, glycolytic and oxidative system). We hypothesized that HIW—as compared to a low-intensity warm-up (LIW)—would increase the proportional contributions from phosphagen and glycolytic systems, and, in doing so, would enhance anaerobic performance as measured by a 100-m sprint time trial.

## 2. Materials and Methods

### 2.1. Participants

Ten young male sprinters with 4.3 years (personal records: 11.57 ± 0.66 s) competitive experience participated in this study (Table S1). The participants’ anthropometric parameters, presented as means ± standard deviation (SD), are as follows: age 16.8 ± 1.1 years, height 175.7 ± 4.6 cm, weight 67.6 ± 4.9 kg, and BMI 21.9 ± 1.0 kg·m^−2^. All participants trained at least five days per week for an average of three hours (18:00~21:00) per day. Each participant provided informed consent after being informed of the study objective and procedure. The study was approved by the Institutional Ethics Committee of CHA University (1044308-202001-HR-001-01). The applied protocols are according to the Declaration of Helsinki.

### 2.2. Study Design

**Figure 1:**
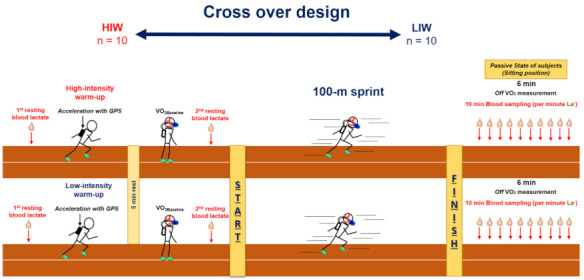
The study design. HIW, high-intensity warm-up; LIW, low-intensity warm-up; *Off* VO_2_, oxygen uptake after 100-m sprint.

The study was conducted in a cross-over design and all participants were randomly assigned to either the HIW or the low-intensity warm-up (LIW) groups (Figure 1). Each session was separated by at least two days and other conditions were kept the same, except for warm-up intensity. Before the warm-up, participants had their first resting blood lactate concentration sampled (mmol·L^−1^; [La^−^]_Rest1_). They completed each warm-up session (either HIW or LIW), and then carried out five minutes of recovery in a standing position to measure resting oxygen uptake (VO_2_). Before the 100-m sprint, each participant had their second lactate concentration measured ([La^−^]_Rest2_). Following this, they performed the 100-m sprint at maximum effort. Participants were rested in the seated position for passive recovery after the 100-m sprint to analyze VO_2_ (6-min) and La^−^ (10-min for every 1-min interval). The study was conducted in the evening (18:00–21:00), which was the participants’ normal training time.

### 2.3. Warm-Up Protocol

**Figure 2:**
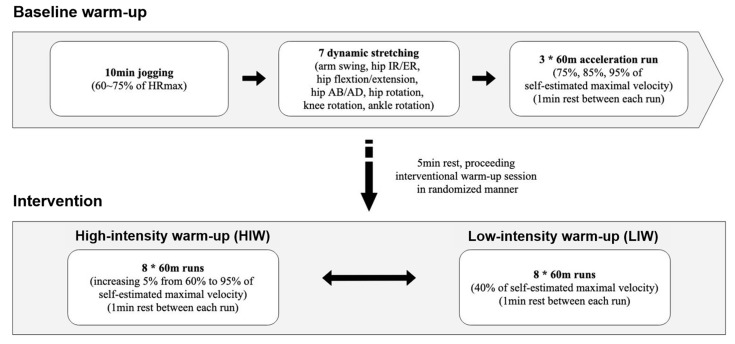
Warm-up protocol. HR_max_, maximal heart rate; IR, internal rotation; ER, external rotation; AB, abduction; AD, adduction.

Each warm-up session was composed of two parts (Figure 2). Both HIW and LIW involved a baseline warm-up (10 min of jogging, dynamic stretching, and three 60-m runs), followed by the interventional warm-up (eight 60-m runs at different intensities [29]. The target heart rate during jogging was set between 117 and 146 beats·min^−1^ (Figure S1), based on calculations using the prediction equation [30]. A five-minute rest was allowed between baseline and interventional warm-ups, and one minute between 60-m runs. In the case of HIW, intervention warm-ups were conducted at 60 to 95% of each individual participant’s self-estimated maximal speed. LIW was performed at 40% of self-estimated maximal speed. Finally, following completion of each warm-up session, athletes rested for five minutes and then performed the 100-m sprint at their maximum capacity.

### 2.4. Study Procedure

Prior to the main experiment, participants visited the laboratory for anthropometric measurement, using a stadiometer (SECA Stadiometer 217, SECA, Hamburg, Germany) and scale (SECA scale 700, SECA, Hamburg, Germany).

Before the warm-up, capillary blood was sampled from the earlobe and placed into heparinized capillaries (20 μL) to analyze the resting blood lactate concentration (mmol·L^−1^; [La^−^]_Rest1_). Blood lactate level was analyzed using an amperometric–enzymatic procedure (Biosen C-line, EKF Diagnostics GmbH, Barleben, Germany).

During the warm-up session, heart rate and top speed were monitored and measured. An H10 (Polar Electro, Kempele, Finland) was used to measure heart rate. Running speed was determined during each 60-m running bout (performed according to the participants’ self-selected capacity) by use of a GPS-based device at 10 Hz (PlayerTek, Catapult Innovations, Melbourne, Australia). Furthermore, top speed was estimated by using the cloud-based analysis software PlayerTek Tam SoftWear (Catapult Innovations, Melbourne, Australia) [31].

**Figure 3:**
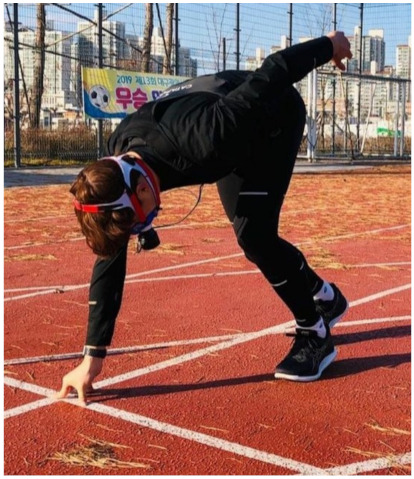
Gas analyzer placement during the 100-m sprint.

Participants had a five-minute rest after all warm-up protocols, and each wore a mobile gas analyzer (Figure 3). The VO_2_ (mL∙kg^−1^∙min^−1^) was measured breath-by-breath using a MetaMax 3B (Cortex Biophysik, Leipzig, Germany). The gas analyzer was calibrated with calibration gas (15% O_2_, 5% CO_2_; Cortex Biophysik, Leipzig, Germany), and the turbine volume transducer was calibrated using a 3-L syringe (Hans Rudolph, Kansas City, MO, USA). Simultaneously, the second lactate concentration ([La^−^]_Rest2_) was measured. The lactate concentration at this time point was also used to the difference, before, and after the 100-m sprint.

The 100-m time trials were assessed by maximal sprint effort and were recorded using an HS-70W (Casio Computer Co. LTC, Tokyo, Japan). Subsequently, participants rested in a seated position for passive recovery without disrupting the accumulated lactate levels in the blood. VO_2_ data were measured for six minutes following the 100-m sprint. In addition, after the sprinting was finished, VO_2Peak_ was extracted from the post-sprint oxygen uptake (VO_2Post_). While the participants recovered, earlobe capillary blood was collected to measure the maximal lactate concentration following the sprint ([La^−^]_Max_) at 10 min for every 1-min interval [5]. The difference in lactate concentrations (ΔLa^−^) was calculated by subtracting [La^−^]_Rest2_ from [La^−^]_Max_.

### 2.5. Calculating Energy System Contributions

To estimate energy demands, the different energy system contributions were evaluated based upon oxygen uptake before (VO_2Baseline_), during (VO_2Sprint_), and after the sprint (VO_2Post_ and ΔLa^−^).

W_Pcr_ was calculated by considering the fast component of excess post-exercise oxygen consumption following the 100-m sprint. W_Pcr_ was determined by subtracting VO_2Baseline_ from the fast component of VO_2Post_. In this study, the VO_2Post_ data were fitted to a mono-exponential model (Equation (1)), because the slow component of the bi-exponential model was negligible. W_Pcr_ was obtained by calculating the integral of the exponential component (Equation (2)) [32,33,34,35].

VO_2(*t*)_ = VO_2Baseline_ + A[e^−(*t*/τ)^] (1)

W_Pcr_ = Aτ (2)

where VO_2(*t*)_ denotes oxygen uptake at time *t*, VO_2Baseline_ represents oxygen uptake before the sprint, *A* is the amplitude, and τ refers to a time constant.

When calculating the W_Gly_, it is assumed that 1 mmol·L^−1^ of ΔLa^−^ corresponds to 3 mL of oxygen uptake per kilogram of body mass [36]. The value of oxygen in milliliters is converted to liters and to energy (kilojoules), assuming that each 1 L of O_2_ is equal to 20.9 kJ [12].

W_Oxi_ was estimated by subtracting VO_2Baseline_ from the area under the VO_2Sprint_. VO_2Baseline_ was obtained in the standing position, from the average of the last 30 s of a 5-min period. Overall, areas under the curve were calculated using the trapezoidal method. The total energy contribution (W_TOTAL_) was estimated as the sum of the three energy systems (W_PCR_ + W_Gly_ + W_Oxi_) in kJ [32,33,34,35].

### 2.6. Statistical Analysis

Statistical analysis was performed using SPSS version 25.0 (IBM corp., Armonk, NY, USA). All results are expressed as mean ± SD. The normal distribution of data was analyzed using the Shapiro–Wilk test. Descriptive analysis was used to indicate participants’ characteristics. A paired t-test and Wilcoxon’s signed-rank test were used to compare variables derived from HIW and LIW of the same participant. Statistical significance was set at *p* < 0.05. The effect size (Cohen’s *d* and Z/√N; *d* and r) was calculated for the parametric and non-parametric tests [37].

## 3. Results

### 3.1. Physiological Parameters

With regard to physiological parameters, no significant differences were observed for VO_2Baseline_, after sprinting VO_2Peak_ and time to VO_2Peak_, between HIW and LIW (*p* > 0.05, *p* > 0.05, and *p* > 0.05, respectively). VO_2Sprint_ in HIW was significantly lower than in LIW (*p* < 0.05, *r* = −0.960) (Table 1).

### 3.2. Blood Lactate Concentration

**Figure 4:**
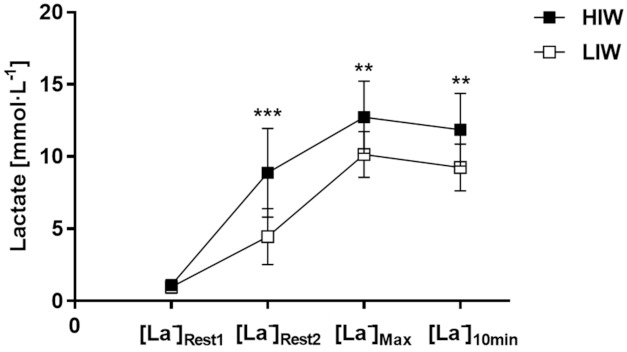
Blood lactate concentration. Mean values (± standard deviation) for HIW (open circles) and LIW (closed squares), before warm-up ([La^−^]_Rest1_), after warm-up ([La^−^]_Rest2_), at the highest lactate concentration after 100-m sprint ([La^−^]_Max_), and for 10th lactate concentration ([La^−^]_10min_), ** (*p* < 0.01), *** (*p* < 0.001).

As shown in Figure 4, no significant differences were observed between HIW and LIW in [La^−^]_Rest1_ (1.1 ± 0.23 vs. 0.98 ± 0.44 mmol·L^−1^, *p* > 0.05). The [La^−^]_Rest2_ exhibited differences between warm-up intensity with higher values in HIW than in LIW (8.88 ± 3.07 vs. 4.45 ± 1.94 mmol·L^−1^, *p* < 0.001, *d* = 1.726). The [La^−^]_Max_ of HIW was significantly higher than for LIW (12.72 ± 2.51 vs. 10.14 ± 1.59 mmol·L^−1^, *p* < 0.01, *d* = 1.227). The [La^−^]_10min_ was significantly higher for HIW (11.86 ± 2.52 mmol·L^−1^) than LIW (9.24 ± 1.61 mmol·L^−1^) (11.86 ± 2.52 vs. 9.24 ± 1.61 mmol·L^−1^, *p* < 0.01, *d* = 1.243).

### 3.3. 100-m Time Sprint Trial

**Figure 5:**
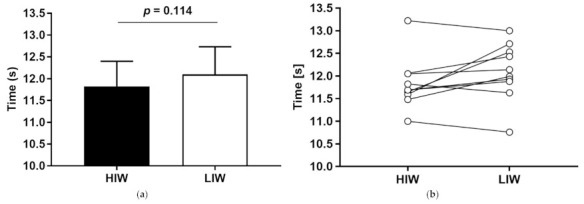
100-m sprint time trial. Mean values (± standard deviation). (**a**) 100-m sprint time trial under different experimental conditions, HIW (closed bar), LIW (open bar), (**b**) Change in individuals’ 100-m sprint time trial.

The 100-m sprint time trial was not significantly different between HIW and LIW (11.83 ± 0.57 and 12.10 ± 0.63 s, *p* > 0.05, respectively) (Figure 5).

### 3.4. Energy System Contributions

**Figure 6:**
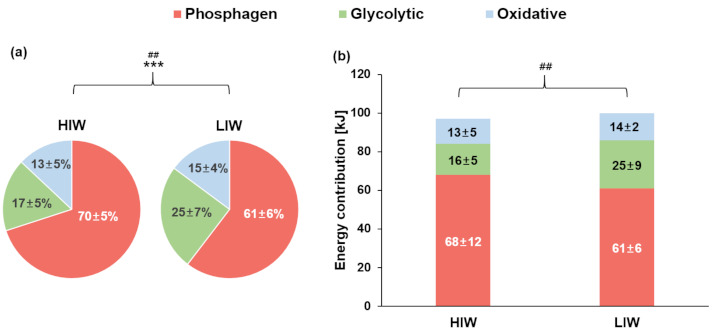
100-m sprint time trial. Mean values (± standard deviation). Estimated relative (**a**) and absolute (**b**) energy system contribution in HIW and LIW. *** Phosphagen system (HIW vs. LIW), ^##^ Glycolytic system (HIW vs. LIW), ^##^ (*p* < 0.01), *** (*p* < 0.001).

Relative (%) and absolute (kJ) energy system contributions are described in Table 1 and Figure 6. For the relative energy demands (%) (Figure 6a), W_Pcr_ was significantly higher in HIW compared to LIW (*p* < 0.001, *d* = 1.592). W_Gly_ was significantly lower in HIW than in LIW (*p* < 0.01, *r* = −0.597), while W_Oxi_ did not show any significant differences between HIW and LIW (*p* > 0.05). For the absolute energy demands (kJ) (Figure 6b), no significant differences were seen between HIW and LIW in terms of W_Pcr_ (*p* > 0.05); however, W_Gly_ was significantly lower in HIW compared to LIW (*p* < 0.01, *r* = −0.627). No significant differences were observed between HIW and LIW in W_Oxi_ (*p* > 0.05). There was no significant difference between HIW and LIW in terms of W_Total_ (*p* > 0.05).

## 4. Discussion

HIW induced a greater contribution from the phosphagen system compared to LIW (approximately 70 vs. 61%). This finding indicates that HIW induced a rapid activation of the creatine kinase reaction, and that substrate-level phosphorylation could be strongly stimulated by a rapid catalysis of high-energy phosphate, phosphorylase kinase, and PFK during a 100-m sprint (i.e., intense short-term exercise) [7,8,14], resulting in an increased proportion of the needed energy being derived from phosphagen. There is evidence to suggest that in order to delay the depletion of the PCr pool during intense muscular contraction, glycolytic ATP can be used simultaneously [38]. Thus, our data suggest that a higher rate of glycolytic enzymatic activity may contribute to an increase in the proportion of phosphagen system utilization during intense activity.

In this study, the glycolytic system contribution under HIW (17%) was lower than that with LIW (24%). However, the [La^−^]_Rest2_ was higher in HIW than in LIW (8.9 ± 3.1 and 4.4 ± 1.9 mmol·L^−1^, respectively). Not surprisingly, ΔLa^−^, which had been calculated from the difference between [La^−^]_Max_ and [La^−^]_Rest2_, was lower in HIW than in LIW, and this ΔLa^−^ difference in HIW contributed to the underestimation of the glycolytic system. These findings are supported by prior studies, showing that ΔLa^−^ value in an HIW (3.8 ± 1.0 mmol·L-1) was markedly lower as compared to reported values [7,15,39]. Our results confirmed that maximal glycolytic capacity ([La^−^]_Max_) was higher in HIW than in LIW (12.7 ± 2.5 and 10.1 ± 1.6 mmol·L^−1^, respectively). Therefore, HIW-induced increases in blood lactate levels implicate the activity of glycolytic enzymes. These results are consistent with those of previous studies, showing increased lactate concentrations are associated with a higher rate of glycolytic enzymatic activity in short-term sprint performance [5,14,15,18,40,41].

In contrast, some researchers have reported conflicting results regarding the effect of HIW on glycolytic system contribution as compared to the current study [42,43]. Wittekind and Beneke [43] did not find a significant difference in Peak [La^−^], despite a strenuous warm-up before the main performance (one-minute sprint cycling), as compared to an easy or moderate warm-up. This conflicting result may be attributed to the duration of the main performance. Previous studies generally found that the effect of HIW evaluated by anaerobic performance testing lasted for at least 15 s [23,24,25,26]. However, Heck et al. [17] reported that short-term maximal sprinting was chosen because it was limited to within 10 s in order to measure the optimized maximal anaerobic capacity. As the elimination of the blood lactate was higher than its production, blood lactate levels might already have been underestimated during the sprint.

The 100-m sprint time trial following the HIW was not statistically different from the times obtained following an LIW, but the participants nonetheless tended to improve their times. Although the 100-m time trial difference was not significantly altered, this finding (−0.27 s in HIW vs. LIW) may be considered valuable given that the shortest race durations are approximately 10 s and the determination of a win or loss occurs in 0.01-s units. Related to the time trial, a higher [La^−^]_Max_ in the HIW appears to support that the HIW optimized the maximal glycolytic capacity during a 100-m sprint [15]. Improved activation of the glycolytic pathway may have an effect in the deceleration phase of a 100-m sprint, with maximal velocity maintained for a relatively longer time following an HIW [4,7].

The effect of an HIW on optimized glycolytic capacity may be more closely related to post-activation performance enhancement (PAPE) than to post-activation potentiation (PAP). PAPE and PAP are warm-up strategies for activating fast-twitch muscle fibers using maximal or near-maximal muscle contraction prior to strenuous performance [44,45,46,47]. The PAP shifts the force–velocity curve upward and to the right through increased calcium sensitivity at filaments and motor unit recruitments of fast-twitch fibers (an increased myosin light chain phosphorylation occurring in type II muscle fibers) [44]. However, increases are also found in the (sometimes maximum) output of voluntary force detected several minutes after high-intensity muscle contractions, which are also most pronounced in muscles with a high proportion of type II fibers [46,47]. Classically, PAP was deemed to reflect this impact.

Although PAP and PAPE are often referred to as the same phenomena, each characteristic shows them to be very different phenomena. Several studies have indicated that each characteristic related to PAP and PAPE differs in the time course of peak voluntary performance enhancement (usually <3 min, half-life ~28 s vs. 6–10 min) after conditioning contractions [29,46,48]. Prieske et al. [47] proposed that the term PAP should only be used to show increases in muscular force/torque production during an electrically evoked twitch, while PAPE should only be used to mention maximal strength, power, and speed after acute muscle activity [46]. The PAPE mechanism may be strongly associated with muscle temperature changes, intramuscular fluid accumulation, and neural mechanisms [46]. In this regard, an increase in muscle temperature may enable an increase in intramuscular energy metabolic components (ATP turnover, PCr utilization rate, anaerobic glycolysis, and muscle glycogenolysis) after intense exercise [44]. Moreover, following high-intensity exercise, increases in blood flow, and subsequently, in intramuscular fluid accumulation, may increase Ca^2+^ sensitivity [46]. Another possibility is that the level of voluntary neural drive to the muscle may increase due to high-intensity events [49].

Our study has some limitations. First, the self-estimated intensity of a warm-up has already been reported to be a method for people who are familiar with physical activity [29]. However, this method is limited in targeting a specific warm-up intensity for speed. To solve the problem, in this study, the top speeds corresponding to each warm-up intensity were measured by a GPS-based device. As a result, the top speeds were gradually increased according to a similar ratio in the HIW, whereas the top speeds following the LIW were steadily maintained (Figure S2). Second, when calculating energy system contribution, the factors that make exact calculations of energy demand difficult can be considered to be mainly due to calculation and measurement errors. Specifically, calculating the W_Gly_ should be determined to a precision that considers measurement errors. In this regard, as is the case for [La^−^] (since this is a concentration), the result depends on the distribution volume, and that may vary for a number of reasons and make precise measurements of energy demands difficult. Furthermore, even the best-performed measurements of oxygen uptake have some errors, with 2–4% often mentioned, and our results may be affected by these factors. Third, a small sample size could influence the statistical power of the data in 100-m sprint performance. Indeed, further performance-related parameters, such as intramuscular data, should be considered in future studies.

## 5. Conclusions

These results indicate that an HIW increases phosphagen and glycolytic system contributions, as compared to an LIW, for the 100-m sprint. Furthermore, a prior HIW for short-term intense exercise has no effect on a 100-m sprint time trial; however, there was the suggestion of a tendency toward improvement. These results show that an HIW is suitable for optimizing anaerobic capacity, and that an HIW may be recommended for the 100-m sprint. These findings suggest that an HIW maximizes rapid activation of the glycolytic system and delays the depletion of skeletal muscle PCr related to the phosphagen system. Both anaerobic energy systems play a crucial role in short-term explosive performance. Therefore, HIW appears to optimize anaerobic energy contribution prior to 100-m sprinting in young male sprinters. Future studies establishing the optimum recovery duration between the HIW and the main performance are necessary.

## Figures and Tables

**Table 1:** Physiological parameters and energy system contributions.

Parameters	HIW	LIW	Significance	Effect Size
	(mean ± SD)	(mean ± SD)	*p*	*r* and *d*
W_PCR_ (kJ)	67.63 ± 12.47	61 ± 6.29	0.232	*r* = 0.285
W_Gly_ (kJ)	15.70 ± 5.30	24.88 ± 8.62 **	0.002	*r* = −0.627
W_Oxi_ (kJ)	12.9 ± 5.13	14.25 ± 2.28	0.460	*d* = 0.340
W_Total_ (kJ)	96.23 ± 13.78	100.2 ± 14.16	0.492	*r* = −0.171
W_PCR_ (%)	69.90 ± 4.80 ***	61 ± 6.29	0.001	*d* = 1.592
W_Gly_ (%)	16.50 ± 5.44	24.60 ± 7.43 **	0.004	*r* = −0.597
W_Oxi_ (%)	13.3 ± 4.52	14.6 ± 3.53	0.488	*r* = −0.160
VO_2Baseline_ (ml∙kg^−1^∙min^−1^)	11.93 ± 3.42	12.51 ± 3.49	0.386	*r* = −0.083
VO_2Sprint_ (ml∙kg^−1^∙min^−1^)	8.41 ± 2.32	10.42 ± 1.84 *	0.014	*d* = −0.960
Post-sprint VO_2Peak_ (ml∙kg^−1^∙min^−1^)	42.48 ± 3.06	44.08 ± 3.53	0.156	*d* = −0.483

W_PCR_, W_Gly_, W_Oxi_: absolute energy contribution from phosphagen, glycolytic, and oxidative system; W_PCR_ (%), W_Gly_ (%), W_Oxi_ (%): relative energy contribution from phosphagen, glycolytic system, and oxidative system; W_Total_: sum of three energy system contribution; VO_2Baseline_: oxygen uptake after warm-up (five-minute rest); VO_2Sprint_: oxygen uptake during 100-m sprint; Post-sprint VO_2Peak_: highest post-sprint oxygen uptake, * (*p* < 0.05), ** (*p* < 0.01), *** (*p* < 0.001).

## Data Availability

Data available on request due to restrictions eg privacy or ethical. The data presented in this study are available on request from the corresponding author. The data are not publicly available due to privacy of CHA University.

## Supplementary Materials

The following information can be found at https://www.mdpi.com/2079-7737/10/3/198/s1. Figure S1. Heart rate during 10 min of jogging at baseline warm-up. Mean values (±standard deviation) for HIW (closed bar) and LIW (open bar). Figure S2. Top speed according to warm-up intensity. Mean values (±standard deviation), Top speed of eight individual 60-m runs during HIW (closed squares) and LIW (open squares). Table S1. PR: personal record.
