# Supplementary material for: High-Intensity Warm-Up Increases Anaerobic Energy Contribution during 100-m Sprint
Source: Biology (Basel). 2021 Mar 5;10(3):198. doi: 10.3390/biology10030198 (PMC7998547; doi:10.3390/biology10030198)
Supplement: Supplementary file 1 [file biology-10-00198-s001.zip › S_Table/S_Table1.docx]

**Table S1.** Athletes' career and personal record.

| **No.** | **Age(years)** | **Career(years)** | **PR(s)** |
| --- | --- | --- | --- |
| 1 | 18 | 5.7 | 11.20 |
| 2 | 16 | 3.8 | 11.80 |
| 3 | 16 | 2.3 | 11.70 |
| 4 | 16 | 3.8 | 11.50 |
| 5 | 18 | 5.3 | 10.41 |
| 6 | 17 | 6.9 | 11.60 |
| 7 | 19 | 1.0 | 12.03 |
| 8 | 16 | 6.8 | 11.25 |
| 9 | 16 | 3.9 | 12.96 |
| 10 | 16 | 3.7 | 11.21 |

PR: personal record
