# Supplementary figures and images for: High-Intensity Warm-Up Increases Anaerobic Energy Contribution during 100-m Sprint
Source: Biology (Basel). 2021 Mar 5;10(3):198. doi: 10.3390/biology10030198 (PMC7998547; doi:10.3390/biology10030198)

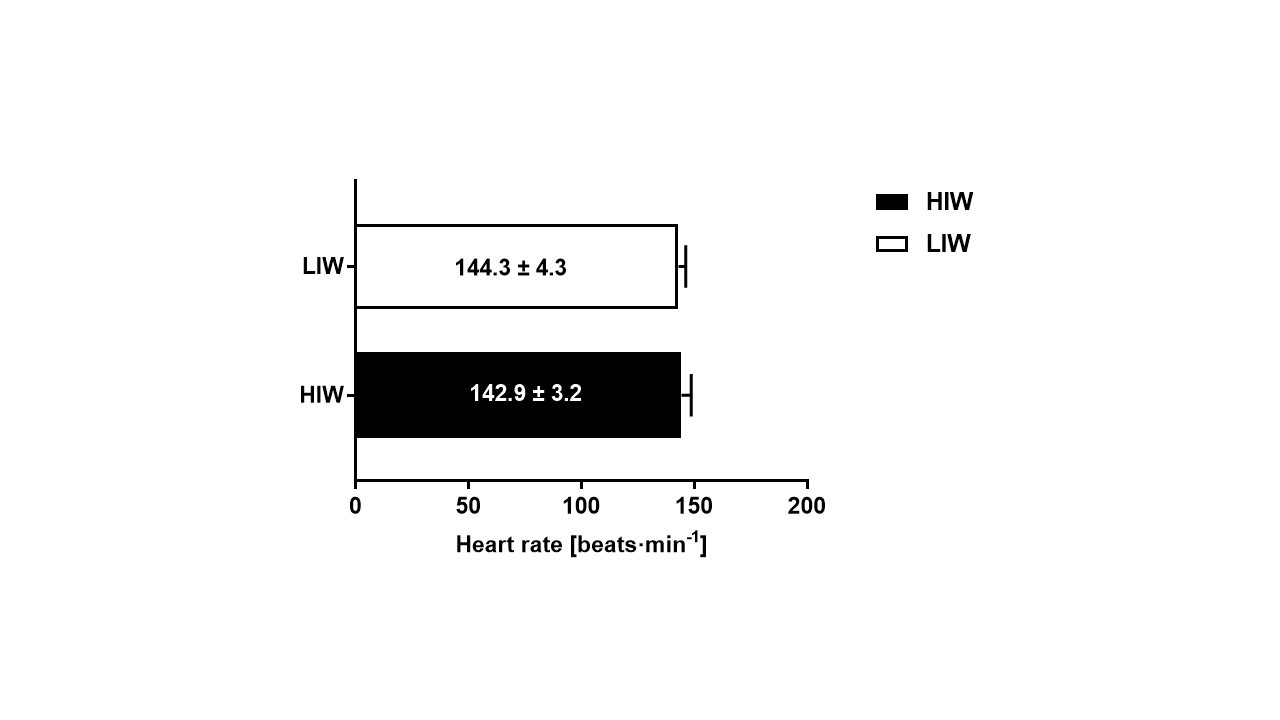

Supplement: Supplementary file 1 [file biology-10-00198-s001.zip › S_Figure/FigureS1.tif]

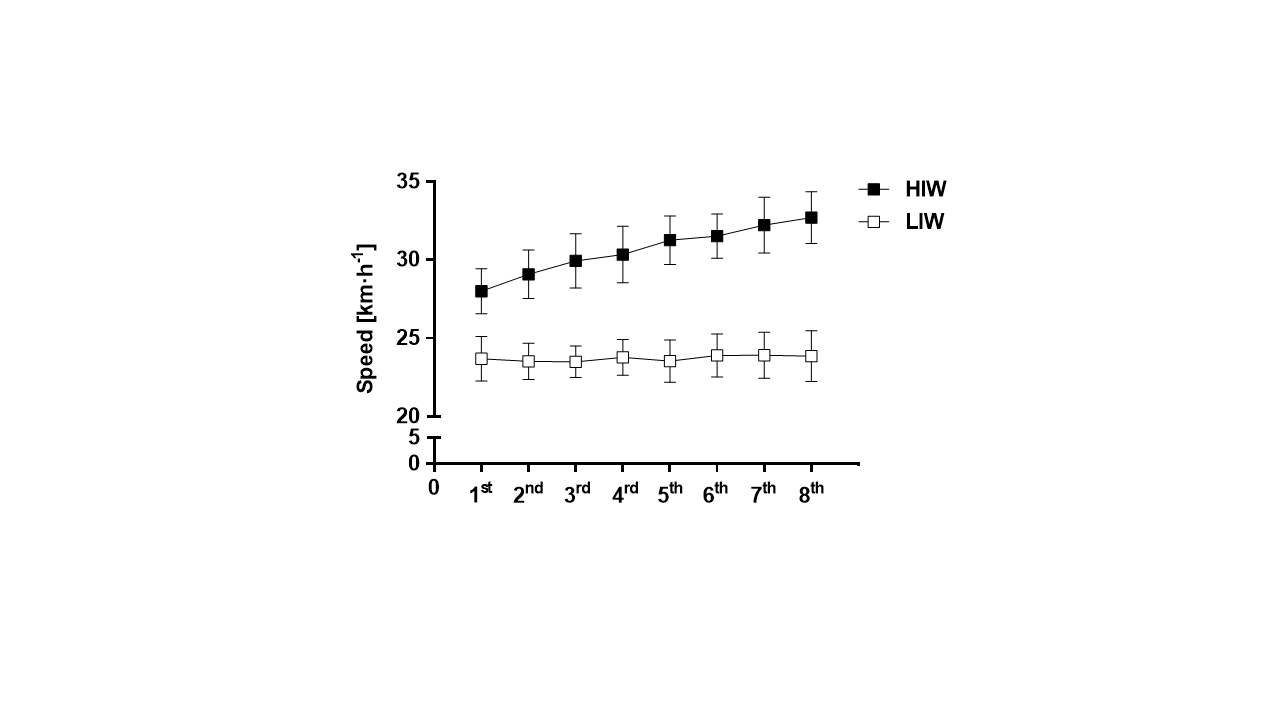

Supplement: Supplementary file 1 [file biology-10-00198-s001.zip › S_Figure/FigureS2.tif]
